# Scientific aspirations of the Chinese Space Station program: an interview with Ming Gao

**DOI:** 10.1093/nsr/nwab161

**Published:** 2021-08-26

**Authors:** Weijie Zhao (赵维杰)

**Affiliations:** NSR, news editor based, Beijing

## Abstract

On 17 June 2021, China's spacecraft Shenzhou 12 took three Chinese astronauts into the Earth's orbit and docked with the assembly of Tianhe and Tianzhou, the core module and cargo ship of the Chinese Space Station (CSS), which had been launched and assembled in orbit earlier this year. The three astronauts became the first visitors of the CSS and would stay in orbit for about three months. In 2022, two laboratory cabin modules, Wentian and Mengtian, will be launched and assembled into Tianhe, completing the basic structure of the CSS. The CSS is designed to be used for at least 10 years, and will provide an outer space experimental platform for researchers from multiple disciplines.

On 23 July 2021, *National Science Review* (*NSR*) interviewed Professor Ming Gao (高铭), the Commander-in-Chief of the Space Utilizaiton System of the China Manned Space Program, Director General of the Technology and Engineering Center for Space Utilization (CSU) of the Chinese Academy of Sciences and an Academician of the International Academy of Astronautics. The Space Utilizaiton System is the scientific part of the CSS project, responsible for the design and organization of the onboard research programs. Gao has been involved in China's Manned Space Program since 1994, and has experienced the whole development process from the Shenzhou spacecraft to the CSS. In this interview, she introduces the scientific goals, plans and aspirations of the CSS.

## EXPERIENCES FROM MIR AND ISS

***NSR:*** Thousands of experiments have been conducted on the Russian Mir space station and the International Space Station (ISS) over the past few decades. What kinds of experiments have been carried out? What were the major achievements?

***Gao:*** Mir operated in orbit for 15 years from 1986 to 2001. According to the official reports, there were more than 100 astronauts who worked on it and more than 1700 experiments conducted. On Mir, the Soviet Union, Russia and some other countries did a lot of work on human physiology, life sciences, materials science and Earth observation, and made significant achievements in biomedical sciences and new materials synthesis, as well as mineral resources exploration.

On the ISS, scientists have done even more experiments, covering more disciplines, including life sciences, microgravity physics, space observation and Earth observation, as well as the development and verification of new technologies. To date, there have been more than 3600 experiments conducted on the ISS, with more than 3000 high-level research articles published, and many patents obtained.

Specifically, on the ISS, researchers made major scientific discoveries relating to organisms’ responses to the microgravity environment, the steady burning of cool flames, Bose-Einstein condensation and dark matter detection; they made breakthroughs in the technologies of space 3D printing, space robotics and space-Earth laser communication; and some of their achievements have already been applied on the ground, such as drugs for osteoporosis and other diseases, artificial

muscle and other new materials, as well as the measurement of carbon dioxide content on the Earth.

Actually, the research programs and achievements of the ISS have provided a valuable reference for the scientific planning of the CSS.

**Figure:**
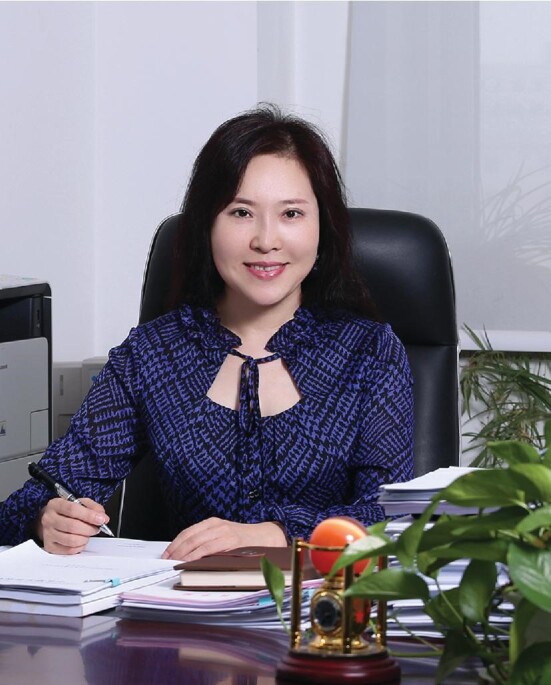
Professor Ming Gao is the Commander-in-Chief of the Space Application System of the China Manned Space Project *(courtesy of Prof. Gao)*.

***NSR:*** I suppose these experiments can benefit human society in at least three aspects: deepen our understanding of the basic physical and cosmological laws, improve our life on Earth and prepare us for future deep space explorations.

***Gao:*** Human activities in space include three closely related aspects: scientific exploration, technological development and practical application.

Scientific exploration in space aims to study fundamental scientific problems such as the origin and evolution of the universe, or the basic laws of matter. This will deepen our understanding of the natural world, and at the same time propel the development of technologies. As an example, the discovery of the steady burning of cool flames will help us to improve fuel engines and reduce pollutant emission.

The direct goal of the development of technology in space is to improve humanity's ability to live in and explore space. On the other hand, these new technologies can be applied on Earth and improve our quality of life. For instance, the technology of space-ground laser communication laid the foundation for the global high-speed communication network.

Space application directly serves both ground application and human's space activities. For example, Earth observation from space has played a major role in monitoring global climate change, and in protecting our marine and terrestrial environment.

## THE SCIENTIFIC ASPIRATIONS OF THE CSS

***NSR:*** What experiments will be performed on the CSS?

***Gao:*** In our planning, inside the CSS cabin we will have more than 20 experiment racks. On the outer side of the CSS, there will be two exposure test platforms and three large payload hanging sites. Moreover, the Chinese Space Station Telescope (CSST) will be launched in 2024. This optical observatory will share the same orbit as the CSS, keeping a certain distance from it; and every two or three years, or when it's needed, it would dock with the CSS for refuelling, maintenace or upgrading.

We made a systematic long-term research plan for four major disciplines, which are life sciences and human body research, microgravity physics, space and earth sciences, and space technologies and applications. Within these four disciplines, we will develop new devices and perform more than 1000 experiments.

Life sciences and human body research on the CSS will study how the cells, organs and tissues react to the space environment and the underlying mechanisms, explore developmental and reproductive processes of plants and animals in space, develop methods for maintaining human health during long-term space flight, and invent novel drugs and medical technologies utilizing the special environment in space.

Microgravity physics aims to learn the basic laws of nature. A high-precision temporal frequency system will be built to verify the general theory of relativity, and to measure the global gravitational field. Researchers hope to achieve Bose-Einstein

Within these four disciplines, we will develop new devices and perform more than 1000 experiments.

—Ming Gao

condensation at an ultralow temperature of 100 pK, in order to perform basic research in ultracold atom physics and low temperature quantum phase transition. They will also study multiphase flow, heat transfer during phase transformation, the characteristics and mechanisms of combustion, and develop new materials.

In the discipline of space and Earth sciences, researchers will make full use of the CSST, the high energy cosmic radiation detection facility and other equipment to perform a variety of cosmological observations. They will study dark matter, dark energy, the origin of cosmic rays, the beginning and early evolution of the universe, and other fundamental problems. They will try to develop new technologies and systems for Earth observation, in order to monitor climate change and to aid global efforts in sustainable development.

For space technologies and applications, we will develop technologies of in-orbit manufacture and construction, space robotics and automated systems, and space information collection and precision measurement, in order to improve humanity's ability to explore and utilize space.

***NSR:*** How were these research projects proposed and approved? Is it a top-down or a bottom-up process?

***Gao:*** It's a combination of both. We first set up the expert committees of the major disciplines. The experts cooperate to

propose an overall plan, and the committees then release announcements to solicit proposals from scientists throughout China. Submitted proposals would go through several rounds of scientific reviews, and be integrated and refined into a series of research plans. This is followed by the verification of engineering feasibility. After rounds of discussions and refinement, we obtained the final Research and Engineering Plan for the CSS project.

Importantly, the research programs will be continuously updated. New programs will be initiated every two to three years. The selected programs would enter our ‘program pool’—the already feasible ones would be approved and implemented, and those not ready yet would undergo preliminary studies for one or two years, during which time the research designs and experimental methods may be further improved, so as to guarantee the quality of the final space experiments.

***NSR:*** China is constructing a ground research center for the CSS, which can be used for the preliminary studies you mentioned. How is the construction of this center progressing?


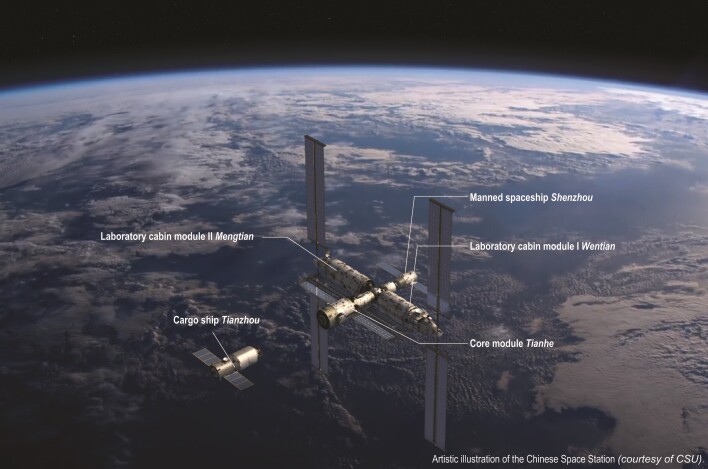


***Gao:*** The center is being constructed in Huairou Science City in Beijing. It is almost completed and is likely to be put into use at the end of this year. The ground center is important not only for preliminary studies; it will play an important role before, during and after the actual space experiments.

Before an experiment is performed in space, a large number of ground experiments are needed to guarantee its feasibility. The ground center can provide experimental conditions similar to that of the space station, enabling scientists to test their experimental settings. During the space experiments, scientists can perform mirror experiments on the ground for comparison; and after the space experiments, they can perform further studies in the center to verify and extend the results obtained in space.

With both the CSS and the ground center, we will be able to create a space-ground coordinated open experimental system, providing an ideal research platform for space science.

***NSR:*** When will the two laboratory cabin modules be launched?

***Gao:*** They will be launched in 2022; Wentian before the end of June and Mengtian before the end of September. By that time, the main structure of the CSS will be complete. This structure may be further extended, if needed, in the future.

***NSR:*** The first three astronauts have been in the CSS for more than a month. Have they begun to help with experiments?

***Gao:*** Yes, they have helped with several experiments, such as the test of the high microgravity experimental device. Now we have two experiment racks in Tianhe, one for containerless materials study and the other for high microgravity study. The astronauts will further help with the high microgravity tests, and the sample replacement of the containerless materials device.

***NSR:*** Will there be scientists working in the space station?

***Gao:*** Yes, of course. There are three types of astronauts: pilot astronauts, space engineers and payload specialists.

The first three astronauts can perform human-machine interactive operations such as replacing samples and performing experiments, as we have talked about. They also act as engineers to repair and maintain the instruments. Also, they are responsible for operations outside the cabin, including instrument installation and the maintenance of the CSST.

On the other hand, the payload specialists have scientific backgrounds and their major duty is to do experiments. They can readjust the experiment design according to real-time conditions and better control the research process in space. We have already recruited four payload specialists with backgrounds in different research areas. They are currently being trained in the astronaut center, and will start their missions after the completion of the CSS assembly.

## INTERNATIONAL COOPERATION AND FUTURE PLANS

***NSR:*** Will there be foreign astronauts working in the CSS?

***Gao:*** Yes, this is possible in principle. The China Manned Space Program has always been open to international cooperation. Several countries have expressed an interest in performing their space research projects in China's space station.

***NSR:*** How are these collaborations progressing?

***Gao:*** We have been collaborating for many years with foreign agencies such as the European Space Agency (ESA), the German Aerospace Center (DLR) and the Italian Space Agency (ASI). We had a successful collaboration with the DLR on our Shenzhou 8 spacecraft. Using a shared biological incubator, we conducted 6 DLR experiments, 10 Chinese experiments and a collaborative experiment. In the CSS project we have 10 ongoing collaborative projects with ESA; scientists from China and Europe will utilize resources from both sides to perform joint research.

In 2018, together with the United Nations Office for Outer Space Affairs, we released an international announcement calling for research proposals from scientists throughout the world. We received 42 proposals from 27 countries, from which we jointly selected 9 proposals as the first batch of international collaborative projects. As with the domestic projects, we will call for new international proposals every two or three years.

***NSR:*** What are the future plans of the China Manned Space Project?

***Gao:*** First of all, the CSS will run for at least 10 years to around 2035. In addition, we are now performing an in-depth assessment of the manned lunar exploration project. In the not-too-distant future, our astronauts will set foot on the moon, and in the long run, we plan to build a lunar base. In the more distant future, we also hope to explore deeper space.

***NSR:*** Do you have any recommendations for the China Manned Space program?

***Gao:*** The China Manned Space program has been developing for nearly 30 years, since the 1990s. During these years, we established a complete project organization and management system, as well as a technological quality control system. I think in the future, we should put more emphasis on the pursuit of scientific problems, and the engineering process should be better adapted to scientific pursuits. Now the CSS has entered a stage of utilization, the significance of scientific goals should receive more attention.

